# Synthesis of Visible-Light-Responsive Cu and N-Codoped AC/TiO_2_ Photocatalyst Through Microwave Irradiation

**DOI:** 10.1186/s11671-016-1503-9

**Published:** 2016-06-13

**Authors:** Fei Tian, Zhansheng Wu, Yujun Yan, Bang-Ce Ye, Dandan Liu

**Affiliations:** School of Chemistry and Chemical Engineering, Shihezi University, Shihezi, 832003 People’s Republic China

**Keywords:** N–Cu-codoped, TiO_2_, Sol-gel, Microwave irradiation, Formaldehyde

## Abstract

N–Cu-activated carbon (AC)/TiO_2_ nanoparticles were prepared by the sol-gel technique through microwave irradiation to modify the visible-light response of TiO_2_. Their structure, surface chemical composition, and optical absorption properties were characterized. The results showed that the codoped particles had a higher surface area and smaller particle size than pure AC/TiO_2_ and monodoped AC/TiO_2_. X-ray photoelectron spectroscopy of N–Cu-AC/TiO_2_ showed that Cu atoms replaced Ti atom sites, whereas N atoms occupied the O atom sites and interstitial sites in the TiO_2_ lattice, which changed the electric and band-gap structures of the photocatalyst. N or Cu monodoping of AC/TiO_2_ reduced the energy band gap of TiO_2_ from 2.86 eV to 2.81 or 2.61 eV, respectively. In (N, Cu)-codoped AC/TiO_2_, N and Cu were incorporated into the TiO_2_ framework and narrowed the band gap of TiO_2_ to 2.47 eV, causing a large red shift and enhancing visible-light utilization efficiency. Photocatalytic activities were further examined by formaldehyde degradation under visible-light irradiation. N–Cu-AC/TiO_2_ was found to have the highest activity (ca. 94.4 % formaldehyde degradation efficiency) and to be easily recyclable. These results show an important and innovative method of improving AC/TiO_2_ activity by modifying the nonmetallic and metallic species.

## Background

Nanocrystalline TiO_2_ has potential for application to photocatalytic degradation of harmful pollutants dispersed in the environment because of its relatively low cost, nontoxicity, favorable optoelectronic properties, and excellent chemical stability [1, 2]. However, the photocatalytic activity and utilization efficiency of visible light are limited because of the small specific surface area and large band gap (3.2 eV) of pure TiO_2_ [3–5]. Adsorption can be increased by preparing a porous material-loaded TiO_2_ photocatalyst, and this has drawn much attention. Given that activated carbon (AC) has a large specific surface area, the reaction rate constant of AC/TiO_2_ is high for the photocatalytic degradation of organic pollutants [6]. In the work of Pastravanu et al., 92 % methyl orange conversion was achieved after 170 min irradiation using AC/TiO_2_ with a large surface area of 357 cm^2^/g [7].

TiO_2_ doping with transition metals has been widely investigated to modify the band gap of TiO_2_ to absorb visible light. Nagaveni et al. [8] reported that Cu^2+^-, V^5+^-, Fe^3+^-, and Zr^4+^-doped TiO_2_ exhibited improved performance in the photodegradation of 4-nitrophenol compared with commercial TiO_2_. Among the various metals doped into TiO_2_, Cu has been considered the most important because of the narrow band-gap energies of its oxides (cupric oxide, CuO, possesses 1.4 eV and cuprous oxide, Cu_2_O, possesses 2.2 eV) and their large light absorption coefficients [9–12]. In addition, Cu doping can reduce the band gap of TiO_2_ to an appropriate value for visible-light adsorption and can reduce the electron-hole recombination rate during photocatalysis. Wang et al. [13] reported that a Cu-doped TiO_2_ thin film exhibited much improved photocatalytic activity compared with pure TiO_2_ thin film in the degradation of a 10 mg/L methylene blue solution under simulated solar-driven irradiation. In recent years, significant efforts have been made to dope TiO_2_ with nonmetallic anions, such as N, S, and C, all of which replace O in the TiO_2_ lattice to generate energy levels just above the top of the TiO_2_ valence band [14–16]. In terms of performance, nitrogen doping is undoubtedly the most impressive solution for improving the visible-light response of TiO_2_ [17–20]. Recently, the concept of second-generation TiO_2_-based materials has been introduced, wherein codoping with two dopant elements produces a synergistic effect to enhance the visible-light absorption efficiency and reduce the recombination processes of the photogenerated charges [21–24]. A Ag/N-codoped TiO_2_ system was prepared using a sonication-assisted sol-gel method by Mothi et al. [25] who found that the system was highly active for the photoconversion of 9-(N,N-dimethylaminomethyl) anthracene.

Microwave-assisted preparation of catalytic materials is gaining increased attention [26]. This method is an efficient alternative because it allows swift heating to the required temperature and extremely rapid rates of crystallization, leading to simplification of the preparation procedure [27–29]. However, codoping Cu and N to TiO_2_ to reduce its band gap and load it onto the AC through a microwave-assisted method has rarely been reported. Thus, an important and innovative method has been attempted to enhance the photodegradation activity of AC/TiO_2_ with visible-light response via modification with Cu and N through microwave irradiation.

In the present paper, AC/TiO_2_, N, Cu-monodoped AC/TiO_2_ and N/Cu-codoped AC/TiO_2_ nanoparticles were synthesized by a sol-gel method under microwave assistance. The phase structure, morphology, specific surface area, and optical properties were investigated using a variety of techniques. Studies on the use of catalysts for photodegradation of HCHO in aqueous solution under visible-light irradiation are also in progress. The concept used in this research can be further applied to modify other materials for improved photocatalytic performance.

## Main Text

### Experimental Methods

#### Catalyst Preparation

AC was prepared by the means described in a previous study [30]. The obtained AC samples were pretreated by addition to HNO_3_ solution, then being left for 24 h. The mixture was filtered using distilled water until it became neutral. The pretreated AC was then dried and stored until use. All reagents were of analytical grade. The TiO_2_ gel/sol was obtained by the conventional sol-gel method [31]. In typical synthesis process, 30 mL of tetrabutyl orthotitanate (TBOT) was dissolved in anhydrous alcohol (EtOH) in proportion of 1:1 (volume ratio). This solution was thoroughly stirred for 40 min and named solution A. Solution B was prepared by mixing 14 mL of glacial acetic acid and 7 mL of distilled water in 35 mL of absolute alcohol. Solution B was added to solution A dropwise and continuously stirred for 1 h. A clear, pale-yellow TiO_2_ sol was then obtained. Pretreated AC (10 g) was added to TiO_2_ sol (100 g). The mixture was placed in an air-dry oven at 100 °C for 24 h. After solidification, AC/TiO_2_ was prepared under microwave irradiation at 700 W for 15 min. To prepare Cu-doped AC/TiO_2_, 0.44 g of Cu(NO_3_)_2_ was mixed with solution B, while for N-doped AC/TiO_2_, 1.71 g of urea was dissolved in solution B. The doped N and Cu in the samples of N-AC/TiO_2_ and N–Cu-AC/TiO_2_ were 0.04 g and 0.01 g, respectively. For N, Cu-codoped AC/TiO_2_, the sample was noted as 0.04 N-0.01 Cu-AC/TiO_2._

### Catalyst Characterization

The crystal structures of the prepared samples were measured by X-ray diffraction (XRD) on a Rigaku D/Max-2500/PC powder diffractometer. Each sample was scanned using Cu-*Kα* radiation with an operating voltage of 40 kV and an operating current of 200 mA. The surface micromorphology of the photocatalyst was characterized by scanning electron microscopy (SEM; S4800, Hitachi Ltd.) at an accelerating voltage of 15 kV. Transmission electron microscopy (TEM) was performed on a Tecnai G2 F20 microscope at 100 kV. FTIR spectra were recorded with a Bruker Vertex FTIR spectrometer, resolution of 2 cm^−1^, in the 4000–400-cm^−1^ range by the KBr pellet technique. UV-vis diffuse reflectance spectra were obtained with a powder UV-vis spectrophotometer (U-4100, Hitachi Ltd.). The specific surface area (SBET, m^2^/g) was calculated using the Brunauer–Emmett–Teller (BET) equation. X-ray photoelectron spectroscopy (XPS) analysis of the samples was conducted using a PHI5700 ESCA system equipped with an Mg *Kα* X-ray source (1253.6 eV) under a vacuum pressure <10^−6^ Pa. The formation rate of ⋅OH at the photo-illuminated sample/water interface was detected by photoluminescence (PL) using terephthalic acid (TA) as a probe molecule. PL spectroscopy of the synthesized products was undertaken at room temperature on a Hitachi F2500 spectrofluorometer using a Xe lamp with an excitation wavelength of 325 nm.

### Photocatalytic Activity

The photocatalytic activity of prepared photocatalyst was measured by degrading the HCHO solution. In a typical test, 50 mg of catalyst was added to 50 mL of HCHO solution (30 mg/L, pH = 6.8). The mixture was then irradiated under a 500 W Xe lamp (any irradiation below 420 nm was removed by using a cut-off filter) to degrade HCHO. The distance between the reactor and lamp housing was 8.5 cm. The concentration of HCHO was measured using an ultraviolet visible spectrophotometer under the maximum absorption wavelength of 413 nm. The removal efficiency, *η*, of the photocatalyst can be calculated as follows:

$$ \eta =\frac{C_o-{C}_t}{C_o}\times 100\%, $$

where *C*_o_ and *C*_t_ are the concentrations of HCHO at initial and different irradiation times, respectively.

The rate of ·OH in the photocatalytic reactions was evaluated by adding 50 mL of terephthalic acid in a manner similar to the above photodegradation experiment. For terephthalic acid, to ensure solubility, solutions were prepared in dilute (2 × 10^−3^ mol/L) NaOH solution. Photocatalyst in the solution was magnetically stirred and illuminated under visible-light irradiation. The photocatalytic experiment was carried out in the presence of 2 mL (10.66 mol/L) t-BuOH, a radical scavenger [32].

## Discussion

### XRD and BET Characterization

**Fig. 1:**
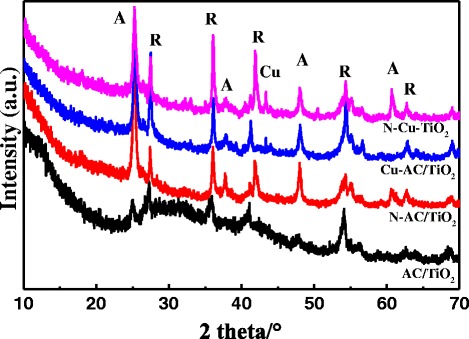
XRD patterns of AC/TiO_2_, N-AC/TiO_2_, Cu-AC/TiO_2_, and N–Cu-AC/TiO_2_ (*A* antase, *R* rutile)

The XRD patterns of the different samples are presented in Fig. 1. The peaks observed at 25.3°, 38°, and 48° represent the anatase crystalline phase, and the peaks at 27.42°, 36.2°, 41.3°, and 44.2° represent the rutile crystalline phase. These all agree with previously reported values [5]. It can be seen that the TiO_2_ crystals obtained via the microwave radiation method with only 15 min, which is conducive to stabilize the AC structure and makes for a more energy-efficient process compared with the traditional heating method [7]. In addition, an additional peak at 43.3°, corresponding to a Cu species, was obtained when Cu was codoped, indicating the formation of Cu^0^ [9]. The Cu^0^ was formed according to the following chemical reaction [14]:

$$ \mathrm{C}{\mathrm{u}}^{2+}\hbox{---} \mathrm{T}\mathrm{i}{\mathrm{O}}_2+{\mathrm{e}}^{\hbox{-}}\kern0.5em \circledR \mathrm{C}{\mathrm{u}}^{+}\hbox{---} \mathrm{T}\mathrm{i}{\mathrm{O}}_2 $$

$$ \mathrm{C}{\mathrm{u}}^{+}\hbox{---} \mathrm{T}\mathrm{i}{\mathrm{O}}_2+{\mathrm{e}}^{\hbox{-}}\circledR \mathrm{C}{\mathrm{u}}^0\hbox{---} \mathrm{T}\mathrm{i}{\mathrm{O}}_2 $$

The XRD patterns show that both the monodoped and codoped AC/TiO_2_ can prevent the phase transformation process from anatase TiO_2_ to rutile from occurring, as shown in Table 1. This may be favorable with respect to degradation of the catalyst [33]. The average crystal sizes of the nanoparticles have been obtained from the Scherrer equation:

**Table 1:** Physicochemical properties of different photocatalysts

Samples	Anatase size (nm)	Rutile size (nm)	SBET (m^2^/g)	Band gap (eV)	Ratio of A and R (%)
AC/TiO_2_	17.9	22.9	532	2.86	47/53
N-AC/TiO_2_	17.2	18.6	487	2.81	62/38
Cu-AC/TiO_2_	16.5	20.6	508	2.61	54/47
N–Cu-AC/TiO_2_	17.8	19.7	548	2.47	57/43

$$ D=k\kern0.5em \lambda /\beta \kern0.5em  \cos \theta, $$

where *D* is the average crystallite diameter (nm), *k* is the X-ray wavelength, *θ* is the Bragg angle, and *β* is the line broadening at half the maximum intensity. The mean crystallite sizes of the doped-TiO_2_ are slightly smaller than that of its precursor (Table 1). In the codoped AC/TiO_2_, the entrance of Cu^2+^ (0.72 Å) into the TiO_2_ crystal lattice to substitute Ti^4+^ (0.68 Å) produces strain through lattice distortions [4], whereas when N^−^ replaces O^2−^ ions, it creates oxygen deficiencies in the TiO_2_ lattice. These features allow the rearrangement of Ti^4+^ and O^2−^ ions in the lattice to interfere with the crystallite growth mechanism and phase transformation [14]. The specific surface area of the catalyst also has an important effect on the degradation of organic compounds because of the synergistic effect of AC and TiO_2_ [31]. The BET surface areas of the AC/TiO_2_ before and after doping were measured by N_2_ adsorption/desorption (Fig. 2) at a liquid N_2_ temperature of 78 K, as shown in Table 1. The doping of ions has little effect on the surface area of activated carbon, and the N–Cu-AC/TiO_2_ maintained a large surface area of 548 m^2^/g.

**Fig. 2:**
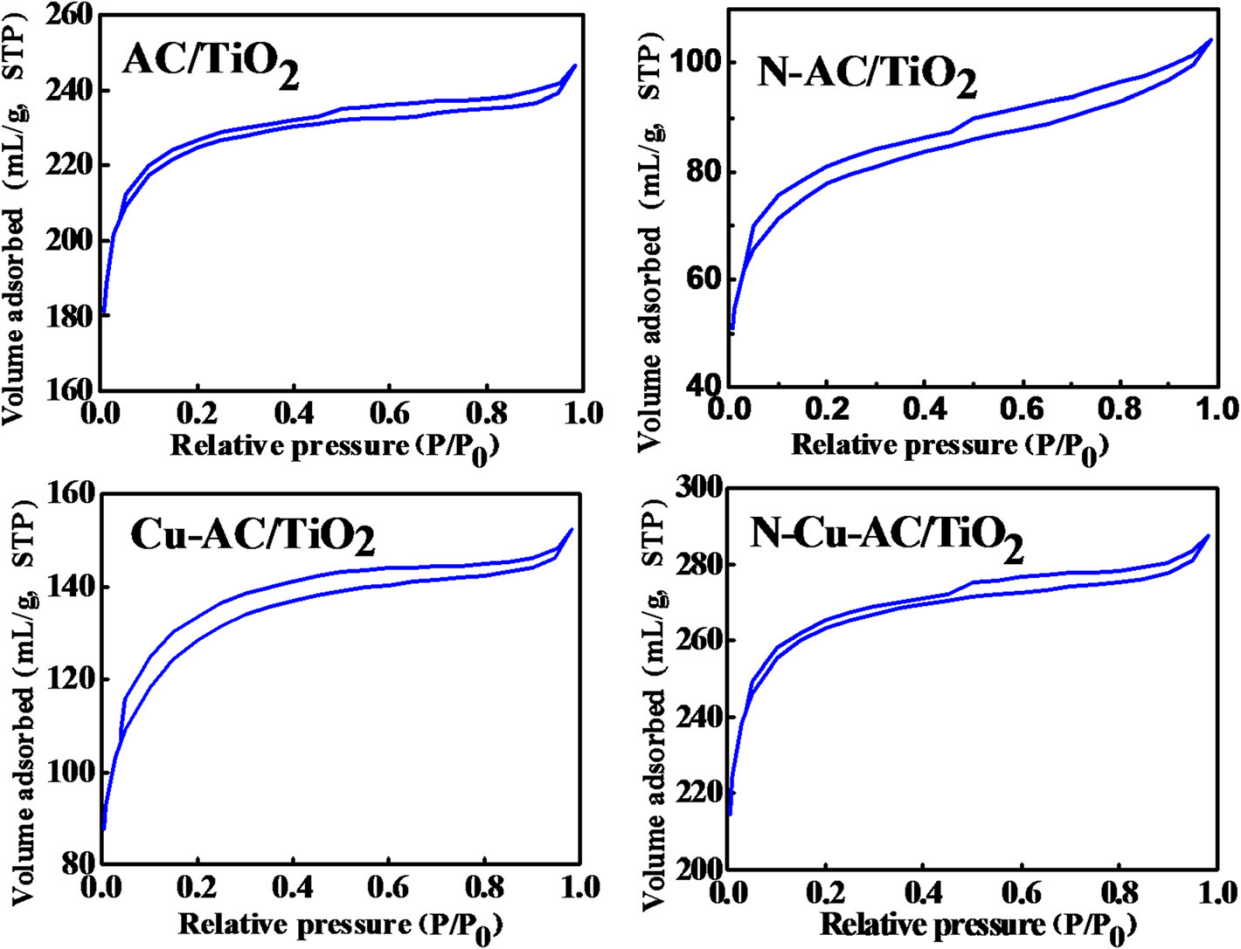
N_2_ adsorption/desorption curves of AC/TiO_2_, N-AC/TiO_2_, Cu-AC/TiO_2_, and N–Cu-AC/TiO_2_

### Morphology of Photocatalysts

**Fig. 3:**
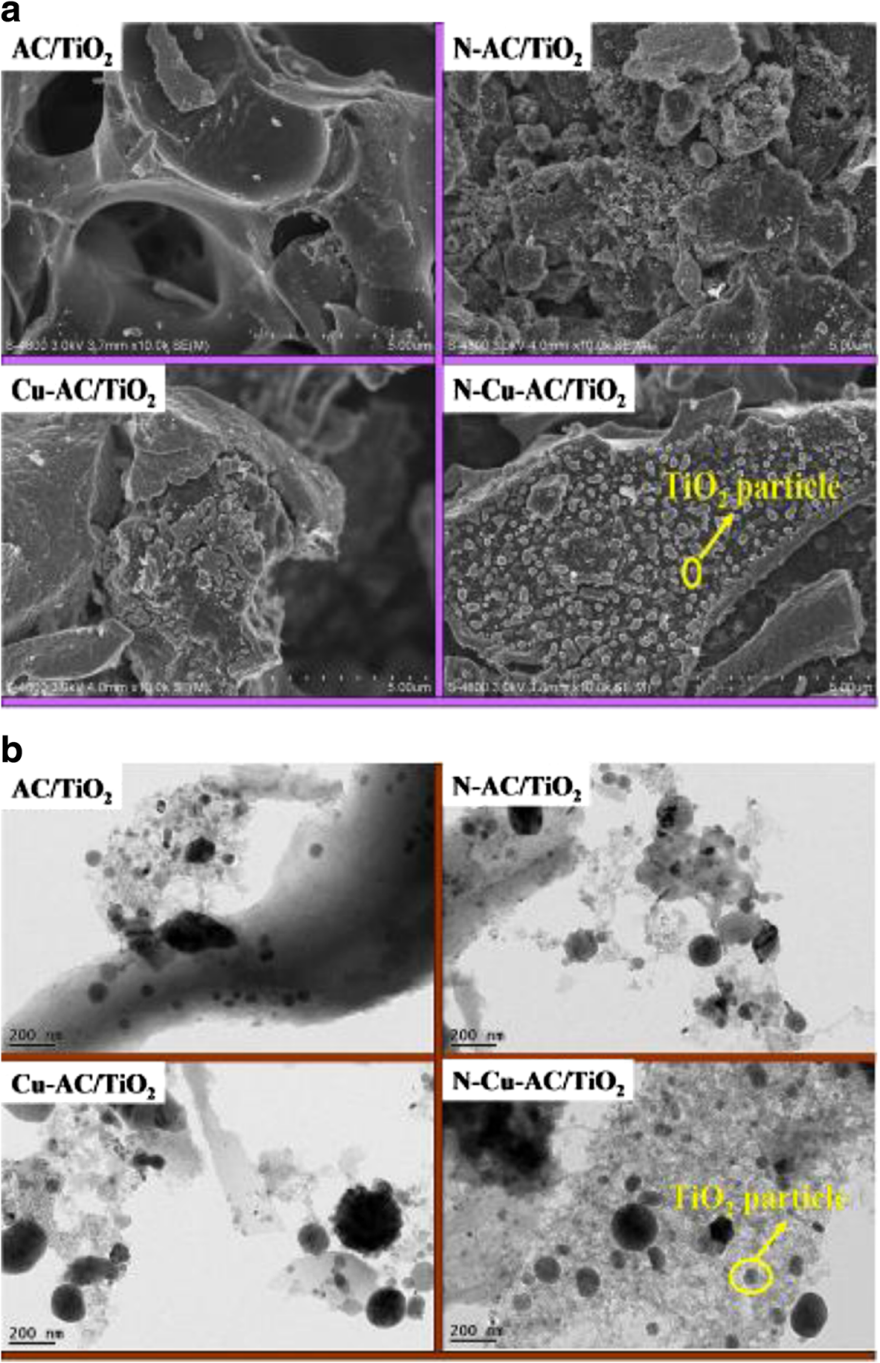
SEM image (**a**) and TEM image (**b**) of different photocatalysts

The surface structure, particle morphology, and crystallite sizes of the photocatalysts before and after doping were investigated by SEM and TEM. SEM images of various photocatalyst powders are presented in Fig. 3a. Undoped AC/TiO_2_ exhibited smooth surfaces with a TiO_2_-particle-like morphology with irregular spherical shapes. Rough surfaces of AC/TiO_2_ spheres with obvious granular features were produced through N or Cu doping. In the case of the N–Cu-AC/TiO_2_ sample, the TiO_2_ particle sizes are quite uniform in a nearly spherical morphology and are well-distributed on the AC surface, as shown in the SEM images. Yi et al. [14] also reported that nonmetal and metal-codoped TiO_2_ present homogeneous morphologies, and this may be beneficial to enhanced catalytic efficiency. The TEM image (Fig. 3b) shows the presence of 10–40 nm powders, consistent with those calculated by XRD (Table 1). However, Cu cannot be observed in the TEM image, suggesting that Cu and N are doped into the TiO_2_ framework. XPS would confirm this.

### FTIR Spectra

**Fig. 4:**
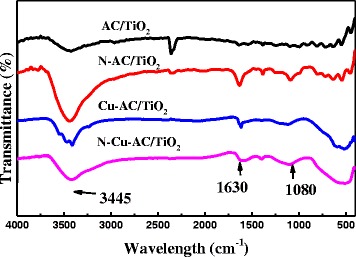
FTIR spectra of AC/TiO_2_, N-AC/TiO_2_, Cu-AC/TiO_2_, and N–Cu-AC/TiO_2_

The FTIR spectra of various photocatalysts are shown in Fig. 4. All samples show similar FTIR spectra, indicating that the TiO_2_ structure did not change after doping with N and Cu. The absorption peak at 3445 cm^−1^ is attributed to the stretching vibration of surface hydroxyl groups—this could yield surface ⋅OH with a high oxidation capability [7]. The vibrations of the hydroxyl moiety were enhanced after AC/TiO_2_ doping (Fig. 4). Thus, doped AC/TiO_2_ may improve photocatalytic activity relative to undoped AC/TiO_2_. A band at around 1630 cm^−1^ is attributed to the bending vibration of the Ti-O bond. Compared with Cu-AC/TiO_2_, N-AC/TiO_2_ and N–Cu-AC/TiO_2_ display an additional peak at approximately 1080 cm^−1^ that can be assigned to the vibration of the N–Ti bond formed when N atoms are embedded in the TiO_2_ network [15].

### UV-vis Diffuse Reflectance Spectroscopy

**Fig. 5:**
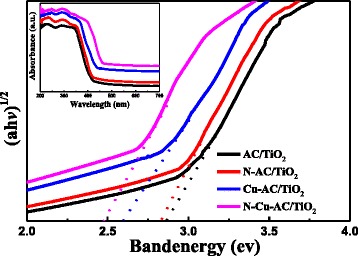
Band-gap energies and UV-vis diffuse reflectance spectra (*inset*) of different photocatalysts

UV-vis diffuse reflectance spectroscopy permits the study of the optical properties and the TiO_2_ framework of the samples. Figure 5 (inset) shows the diffuse reflectance spectra of the prepared photocatalyst samples in the range 200–700 nm. The spectrum for pure AC/TiO_2_ shows an absorption edge of approximately 412 nm, whereas the N, Cu-monodoped samples show a red shift in the absorption edge and a strong absorption in the visible-light region. The absorption wavelengths of N, Cu-codoped AC/TiO_2_ increased to 450 nm. Application of the Kubelka–Munk [8] conversion can explain the red shift:

$$ \alpha h\nu =A{\left(h\nu \mathit{\hbox{-}}{E}_g\right)}^{1/2}, $$

where α, *h*ν, *E*_g_, and *A* are the absorption coefficient, photon energy, band-gap energy, and a constant, respectively. In accordance with the above equation, a modified plot of the Kubelka–Munk function versus the energy of the exciting light was used to determine the band gap of the samples. A band-gap value of 2.86 eV was obtained for AC/TiO_2_. According to data reported in literature, it is well-known that bulk anatase and rutile have the direct band-gap energy of 3.2 and 3.0 eV, respectively [3, 5]. This means that the radiation wavelength used for electron excitation of the titania should be smaller at about 387 nm (UV region). The band gap of TiO_2_-coated on AC samples decreased to 2.86 eV, indicating an influence of the AC support on the optical properties of TiO_2_ photocatalyst. Coromelci-Pastravanu et al. [7] also reported that the optical band gap of TiO_2_-coated mesoporous carbon samples decreases slightly comprised with pure TiO_2_. The carbon-based composites were found to be able to promote a rapid photoinduced charge separation and a slow charge recombination, by accepting photogenerated electrons from the photocatalytic TiO_2_ nanoparticles. After N doping, the band gap of TiO_2_ decreased to 2.81 eV and the band gap of Cu-AC/TiO_2_ was about 2.61 eV. Meanwhile, significant narrowing of the band gap to approximately 2.47 eV was observed after N, Cu codoping. This significant band-gap reduction is associated with charge-transfer, corresponding to the electronic excitation from the valence band (VB) to the conduction band (CB). Reduction in the band gaps correspondingly reduced the energy required for electron transition from the VB to the CB, thus shifting the optical absorption to a lower energy. This large decrease in band gap for the codoped catalyst may be attributed to the formation of mixed energy levels between the VB and CB. Thus, Cu and N doping promotes the visible absorption of the catalyst and plays a significant role in enhancing the photocatalytic activity of the catalysts.

### XPS Characterization

**Fig. 6:**
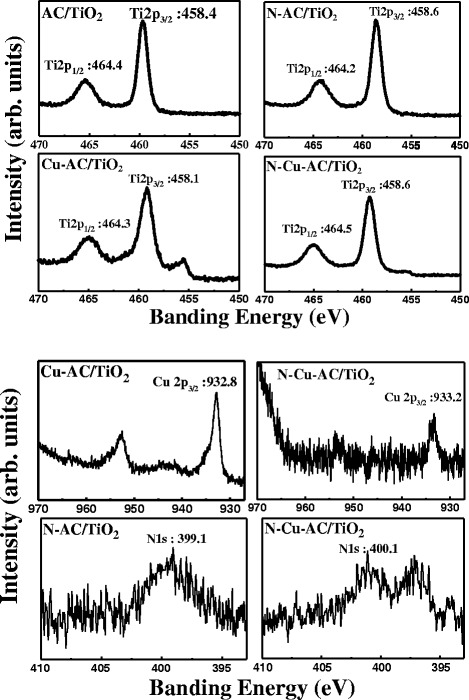
XPS spectra of Ti2p, Cu2p, and N1s levels of various photocatalysts

XPS analysis of different photocatalysts was performed to examine the chemical states of each element (shown in Fig. 6). From the XPS spectra, all samples display peaks at about 458 and 464 eV, and these can be assigned to the Ti 2p_3/2_ and Ti 2p_1/2_ of Ti^4+^ states, respectively, in the Ti2p core level [10]. Thus, the Ti is found as Ti^4+^ in the samples and this was not significantly affected by the incorporation of Cu and N. When investigating the Cu2p core level of Cu-AC/TiO_2_ and N–Cu-AC/TiO_2_, Cu was found to be present only in the oxidation state of Cu(II), with corresponding peaks at 932.8 (Cu-AC) and 933.2 (N–Cu-AC) [9]. This finding signifies that Cu is incorporated into the TiO_2_ lattice, in agreement with the XRD results. The peak at approximately 400 eV can be attributed to the interstitial N atoms in N–O bonds or Ti–(N–O) bonds in the case of N-AC/TiO_2_ and Cu–N-AC/TiO_2_, indicating that some nitrogen groups are adsorbed on the TiO_2_ as the interstitial N-doping [19]. An isolated level is formed above the VB because of the Cu-3d orbital in the case of Cu-doped AC/TiO_2_ [10], and this decreased the energy required for electronic excitation from the VB to CB, in accordance with the UV-vis results. Lin et al. [2] also reported that Cu doping makes the absorption edge of TiO_2_ red shift because of the transition of the excited 3d electrons from the Cu^2+^ ions to the CB of TiO_2_.

### PL Spectra

**Fig. 7:**
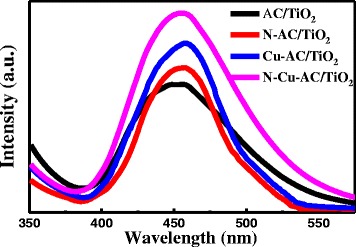
Photoluminescence emission spectra of various photocatalysts

The ⋅OH radicals in the reaction systems were detected by PL (Fig. 7). The radicals were generated on the four samples after illumination. The formation rate of the OH radicals on the AC/TiO_2_ powders increased after Cu, N doping on TiO_2_, and the generation rate of the ⋅OH radicals on the N–Cu-AC/TiO_2_ surface was higher than that of the other photocatalysts. A previous study reported that ⋅OH is the major reactive species for photocatalytic oxidation [33]. Sin et al. [34] also found that ⋅OH was the main active species during the Eu/ZnO degradation of phenol. The production rate of ⋅OH follows the order N–Cu-AC/TiO_2_ > Cu-AC/TiO_2_ > N-AC/TiO_2_ > AC/TiO_2_.

### Photocatalytic Activity of Photocatalysts

**Fig. 8:**
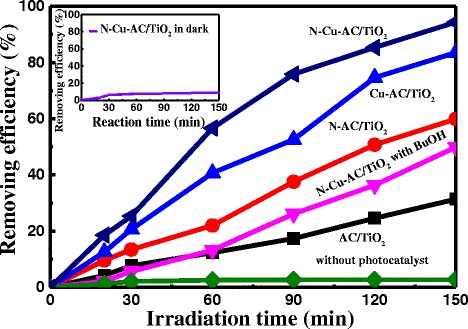
Photocatalytic degradation of HCHO solution by AC/TiO_2_, N-AC/TiO_2_, Cu-AC/TiO_2_, and Cu–N-AC/TiO_2_ and the comparison experiments

The photocatalytic activity of these photocatalysts was evaluated by degradation of HCHO, as shown in Fig. 8. Under identical experimental conditions, N–Cu-AC/TiO_2_ showed much higher activities (94.38 % degradation efficiency) than those of pure AC/TiO_2_ (31.4 % degradation efficiency), N-AC/TiO_2_ (59.9 % degradation efficiency), and Cu-AC/TiO_2_ (83.6 % degradation efficiency), while the removing efficiency of HCHO without photocatalyst under irradiation and with N–Cu-AC/TiO_2_ in the dark for 150 min was about 2.74 and 8.67 %, respectively. First, the photocatalytic activity of the products corresponds to the generation rate of the ⋅OH radicals. Photocatalysis of HCHO in the presence of radical scavengers reduced reaction efficiency from 94.38 to 49.89 %, indicating that the hydroxyl radical initiated degradation is the dominant route over degradation by reactive electron-hole pairs on the catalyst surface and photolytic cleavage. The anatase/rutile ratio is an important factor in determining the photocatalytic activity of the samples through the formation of ⋅OH. The ratio of anatase/rutile (54/47) in Cu-AC/TiO_2_ is greater than that of pure AC/TiO_2_ (47/53). This resulted in more ⋅OH on the Cu-AC/TiO_2_ surface under irradiation. In N, Cu-codoped AC/TiO_2_, the ratio of anatase/rutile increased to 57/43, which led to the highest ⋅OH formation rate of the reaction systems studied. Commonly, the composite of two phases of the same semiconductor is beneficial for reducing the combination of photogenerated electrons and holes [1], thereby enhancing the ⋅OH formation rate. In N-AC/TiO_2_, the formation rate of ⋅OH decreased with the increase in anatase/rutile ratio to 62/38, implying that the optimal anatase to rutile ratio is likely approximately 57/43.

The most important factor for the enhancement of photocatalytic activity of doped AC/TiO_2_ is that Cu substitution of the partial Ti leads to an isolated energy band formation between the VB and CB in the band structure of TiO_2_. The formation of ⋅OH is attributed to the transition of electrons between the VB and CB. When doped with Cu, a two-step optical transition will occur because of the formation of the newly isolated energy band and the decrease in band-gap energy between the VB and the CB of TiO_2_, as shown in Fig. 5. This increased the optical response of Cu-doped AC/TiO_2_. In N-AC/TiO_2_, the Ti–N linkage may lead to formation of an N1s peak because of substitutional N doping in the TiO_2_ lattice [23]. Doping of N into TiO_2_ forms a new state on N1s, just above the VB, leading to strong absorption of visible light and enhancement in the separation efficiency of the photoinduced electrons and holes. The significant enhancement of photocatalytic activity of N, Cu-codoped AC/TiO_2_ in this study can be attributed to the synergistic effect of N, Cu codoping. Doping of the TiO_2_ lattice with Cu can lead to O vacancy production, which in turn facilitates N doping. Both Cu and N doped on TiO_2_ decreases the electronic transition energy and improve the photocatalytic activity of N–Cu-AC/TiO_2_.

### Mechanism of N, Cu-Codoped AC/TiO_2_

**Fig. 9:**
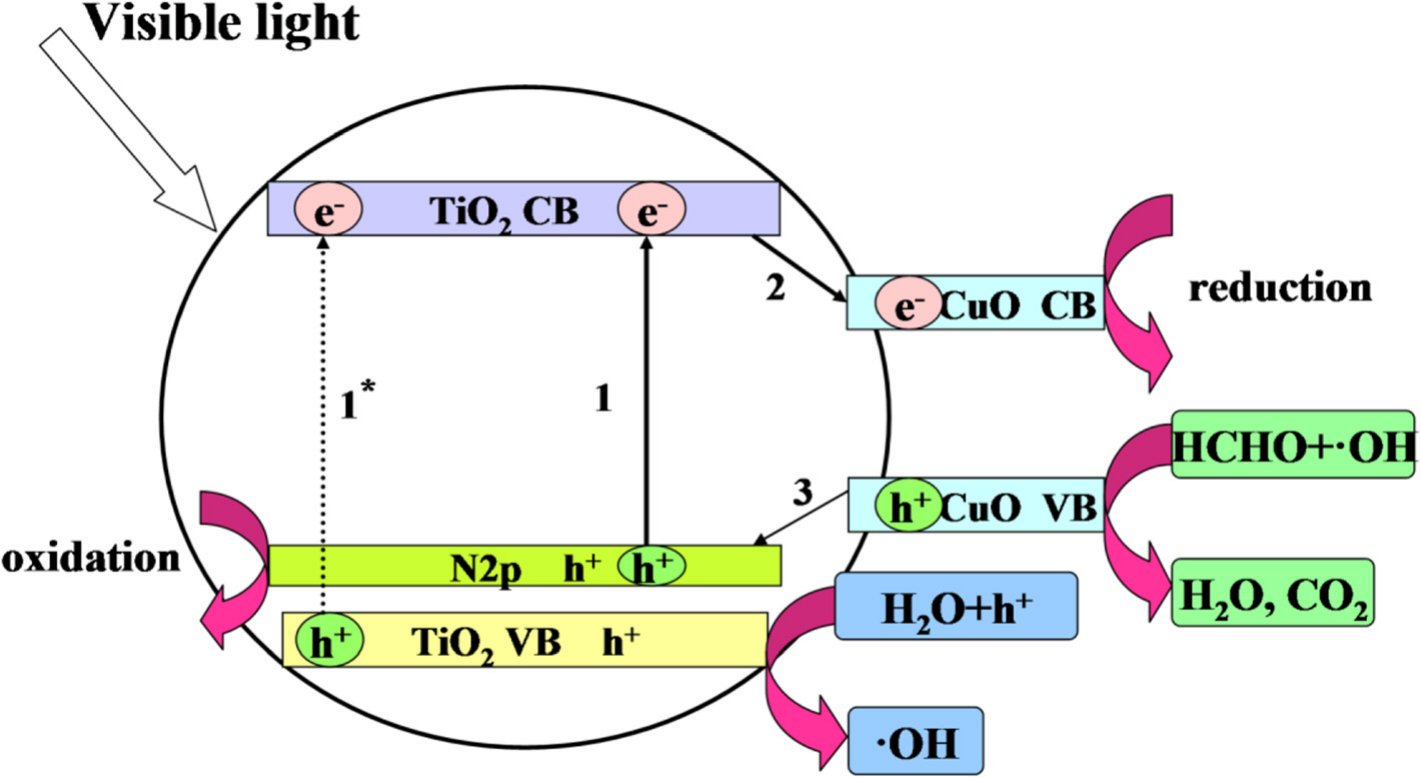
Schematic diagram of band gap narrowing for N, Cu-codoped AC/TiO_2_

Figure 9 illustrates the band structures of the N, Cu-codoped AC/TiO_2_ materials. Possible mechanisms responsible for the observed improvement in photocatalytic activity of the new material under visible-light irradiation are proposed. When nitrogen is incorporated into the TiO_2_ lattice, the N2p states are positioned above the TiO_2_ CB, which increases the CB energy by 0.46 eV [35]. TiO_2_ band-gap narrowing also takes place, as shown in procedure 1 and compared with 1* in Fig. 9. Thus, the response light of N-AC/TiO_2_ is increased to 425 nm compared with 415 nm for pure AC/TiO_2_ (Fig. 5). For the Cu co-modified N-AC/TiO_2_, the light response increased to 465 nm (Fig. 5) because the band energy of TiO_2_ decreased to 2.47 eV (Table 1). In N–Cu-AC/TiO_2_, the Cu d-states lie deep in the band gap that lies below the CB of TiO_2_ [10]. In the case of visible-light irradiation, TiO_2_ can be excited at a wavelength <465 nm (procedure 1). Thereafter, the excited electron may be transferred from the TiO_2_ CB to the Cu CB (procedure 2), thereby improving the charge separation and allowing oxidation/reduction processes to occur on the TiO_2_ VB/Cu CB. Yang et al. [36] also reported that Cu species have smaller band gaps and higher work functions than does bare TiO_2_, so the electron can transfer from the CB of TiO_2_ to a metallic copper ion. The excited electrons can be further scavenged by surface-adsorbed H_2_O on the TiO_2_ to generate ⋅OH and other active species. Moreover, the electron from the Cu VB may also be transferred to the TiO_2_ VB (procedure 3), creating a hole in the Cu VB. The additional charge separation significantly improves the photocatalytic performance. In addition, the mixed phases of anatase and rutile in TiO_2_ can inhibit the electron-hole recombination of TiO_2_ and thus enhance the photocatalytic ability [37, 38]. Thus, the photocatalytic performance enhancement of TiO_2_ may be attributed to the synergistic effect of Cu and N co-implantation, which resulted in a change in the electronic and band-gap structures.

### Reusability of the Catalyst

**Fig. 10:**
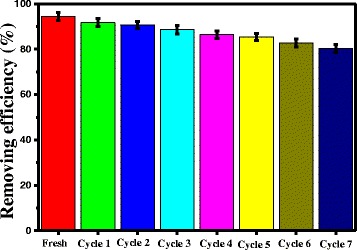
Repetitive use of N–Cu-AC/TiO_2_ photocatalyst under visible-light irradiation

The stability and recyclability of a photocatalyst are important for its potential application. To evaluate the stability of the N–Cu-AC/TiO_2_ catalysts, the nanoparticles were collected after the photocatalytic reaction, and then the nanoparticles were put in ethanol solution with ultrasonic treatment for 2 h, dried at 120 °C for 4 h. The photocatalytic test was repeated up to seven times while keeping all other parameters constant. Figure 10 shows that, after the seven recycling experiments, the N–Cu-AC/TiO_2_ particles retained their high photocatalytic activity, and 80.2 % of the HCHO was degraded. This result indicated that the N, Cu-codoped AC/TiO_2_ prepared in the present study had good photocatalytic stability and allowed for possible repeat utilization.

## Conclusion

A novel and simple technique for incorporating N and Cu on TiO_2_ nanoparticles loaded on AC was presented. The nanoscale TiO_2_ showed anatase and rutile phases with a particle size of 18 nm, an appropriate proportion in codoped AC/TiO_2_, and a specific surface area of 548 m^2^/g. Nitrogen and Cu may occupy the internal TiO_2_ crystal framework, replacing some Ti^4+^ and O^2−^, respectively, and extending the band-gap excitation to the visible region. The obtained N–Cu-AC/TiO_2_ shows a distinctive absorption band in the visible region and presents the lowest band-gap value among all of the samples studied. The N–Cu-AC/TiO_2_ photocatalyst exhibited good photocatalytic activity for the degradation of HCHO under visible-light irradiation. The incorporation of Cu and N may decrease the required energy for electronic transitions, thereby improving the photocatalytic activity. Furthermore, the produced photocatalyst can be easily recycled and exhibits enhanced stability. Therefore, using N, Cu-doped AC/TiO_2_ for pollutant photodegradation is a practical method for purifying water under visible light.
